# No-Touch Saphenous Vein Graft Harvesting to Maintain the Success of CABG: comments on the SUPERIOR SVG Trial

**DOI:** 10.21470/1678-9741-2020-0107

**Published:** 2020

**Authors:** Tomislav Kopjar, Bruno Botelho Pinheiro, Michael Richard Dashwood

**Affiliations:** 1 Department of Cardiac Surgery, University of Zagreb School of Medicine and University Hospital Center Zagreb, Zagreb-1000, Croatia. E-mail: tkopjar@gmail.com; 2 Department of Cardiovascular Surgery, Hospital do Coração Anis Rassi, Goiânia, GO, Brazil.; 3 Surgical and Interventional Sciences, University College London Medical School, London, United Kingdom.

**Dear Editor,**

In the recent interview with Dr. Domingos Souza, published on the *Journal’s* blog, initial observations that lead to the introduction of the no-touch (NT) saphenous vein graft (SVG) harvesting technique for coronary artery bypass grafting (CABG) were discussed^[1]^. Quoting Albert Einstein (“imagination is more important than knowledge”), Souza admits that his 1989 observations during CABG surgery lead to the advent of the NT technique. He observed that during conventional (CON) SVG harvesting, venospasm occurs exactly when the perivascular tissue is removed, hence the idea of keeping the perivascular tissue intact was born.

**Fig. 1:**
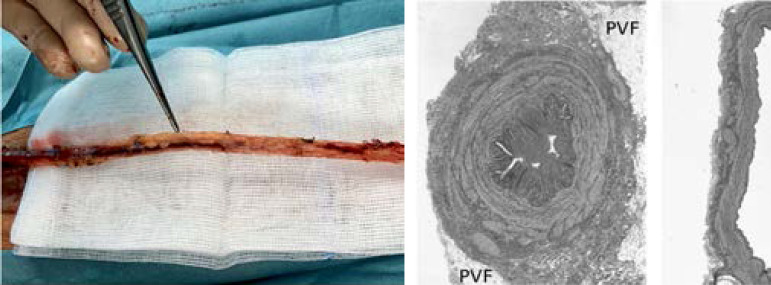
Left panel: intraoperative no-touch saphenous vein graft harvesting. The graft was harvested complete with a pedicle of surrounding perivascular fat, to minimize surgical trauma and thus preserve the normal vein architecture. One side of the vein was consistently marked with dye for easier orientation to avoid twisting. Middle panel: representative transverse section of a no-touch saphenous vein graft with all the vessel wall layers (intima, media and adventitia) undamaged and perivascular fat intact (Dashwood et al.^[6]^, 2009). Right panel: conventional saphenous vein graft showing surgical trauma-induced vascular damage and removal of perivascular fat (Dashwood et al. ^[6]^, 2009).

While arterial grafts are generally harvested with the outer pedicle intact, the SVG, when prepared as described by Favaloro, is harvested with the outer pedicle removed^[2]^. Here, the trauma inflicted to the saphenous vein causes considerable vascular damage, which has a pronounced effect on graft patency. When using the NT technique, the SVG is harvested with minimal trauma and with the outer fat pedicle intact (Figure 1). This procedure not only minimizes vascular damage but also obviates the need for high pressure intraluminal saline distension, which is often used to overcome venospasm^[1]^. Since the introduction of NT harvesting, a number of follow-up studies have been performed with NT SVGs, showing a marked improvement in graft patency when compared with CON SVGs at 16 years^[3]^.

Bypass surgery remains the most common cardiac procedure and demonstrates a survival benefit over percutaneous coronary intervention for complex and left main coronary artery disease. The CABG success is largely based on the long-term patency rate of bypass grafts. Improved long-term results are associated with the use of the internal mammary artery. High occlusion rate is considered the main disadvantage of venous grafts. Nevertheless, SVG is still widely used for CABG. To address the low patency rates of SVG, an atraumatic NT harvesting technique was developed. When using the NT technique, the SVG is harvested with a pedicle of surrounding tissue to minimize surgical trauma and, thus, preserve the normal vein architecture (Figure 1). In spite of the general belief that arterial grafts are superior to venous grafts, NT SVG proved to be an excellent alternative to the radial artery with similar patency at 8 years after CABG^[4]^.

Mechanisms underlying the success of NT SVGs are multifactorial and have been investigated using a number of techniques over the past 25 years^[5]^. Contributing factors range from the preservation of an intact endothelium to the mechanical support provided by the intact surrounding cushion of fat and the beneficial role of adipocyte-derived factors^[6]^. Despite the evidence supporting the benefits of NT SVG harvesting, this technique has not gained popularity among certain surgeons, particularly in the USA. Opponents often emphasize the lack of multicentric data in support of the technique and issues with leg wound healing. The recent SUPERIOR SVG trial is the only international multicenter randomized controlled trial to date that has explored the effects of the two SVG harvesting techniques (NT *vs*. CON) on patency and clinical outcomes one year after CABG^[7]^. We believe that there are some aspects of the SUPERIOR SVG trial that might have had negative impact on the results of the NT group.

In the SUPERIOR SVG trial, adverse events in leg wounds between the two study groups (NT *vs*. CON) were assessed^[7]^. It is well known that endoscopic vein harvesting is associated with a reduced leg wound complication rate when compared to open vein harvesting. Endoscopic vein harvesting was part of the study protocol in the SUPERIOR SVG trial. Including endoscopic vein harvesting in the study protocol would not have been an issue if equally distributed among groups. However, the CON patient group underwent either open or endoscopic SVG harvesting, whereas in the NT group, the SVG was harvested purely with the open technique. This is a source of a major selection bias. Therefore, the validity and reliability of the analysis of adverse events in legs may be a matter of debate. While concerns regarding leg wound complication when using the NT technique have been raised in the past, ongoing attempts aimed at overcoming these problems are in progress. Once further progress is made using minimally invasive no-touch harvesting, the NT SVG should ensure its position as the second conduit of choice for CABG^[8]^.

The main objective of the SUPERIOR SVG trial was to compare the proportion of SVG occlusions in 1 year following CABG^[7]^. Based on the intention-to-treat analysis of the study grafts, the trial failed to show a significant difference in the proportion of graft stenosis or occlusion between groups (NT: 7.8%; CON: 15%; *P*=0.11). In the on-treatment analysis, where both study and non-study grafts were included, graft stenosis or occlusion was significantly reduced in the NT group (NT: 9.9%; CON: 18.9%; *P*=0.018). The explanation for the absence of a patency benefit in the intention-to-treat analysis might be due to the small sample size. Estimates of the study sample size indicated that 615 patients in each group could identify a relative risk reduction of 30% (NT: 14%; CON: 20%) for study graft occlusion, but the study finally included 250 patients overall. In addition, vein harvesting was performed by several trainees and attending surgeons at different levels of training. It would have been interesting to assess the SV structure with histology to explore the degree of vascular damage inflicted on the study grafts by the different harvesters. In addition, the authors confuse the reader by including a pharmaceutical arm in the study, investigating the effect of fish oil and placebo, but does not give data regarding this intervention. Why was this secondary study included, but no data was presented?

In the 2018 ESC/EACTS Guidelines on myocardial revascularization, the NT technique of SVG harvesting was recognized for the first time and received a class IIa recommendation when an open vein harvesting technique is used^[9]^. The SUPERIOR SVG trial, although eagerly awaited, leaves several questions unanswered. Careful conduction and interpretation of such randomized controlled trials are extremely important to maintain their position as the hallmark of evidence-based medicine and the basis for translating research data into clinical practice.
